# Nonlinear robust integral backstepping based MPPT control for stand-alone photovoltaic system

**DOI:** 10.1371/journal.pone.0231749

**Published:** 2020-05-19

**Authors:** Kamran Ali, Qudrat Khan, Shafaat Ullah, Ilyas Khan, Laiq Khan

**Affiliations:** 1 Department of Electrical and Computer Engineering, COMSATS University Islamabad, Abbottabad Campus, Abbottabad, KP, Pakistan; 2 Center for Advance Studies in Telecommunication (CAST), COMSATS University Islamabad, Islamabad, Pakistan; 3 Faculty of Mathematics and Statistics, Ton Duc Thang University, Ho Chi Minh City, Vietnam; Huazhong University of Science and Technology, CHINA

## Abstract

PV (Photovoltaic) cells have nonlinear current-voltage (*I* − *V*) and power-voltage (*P* − *V*) characteristics with a distinct maximum power point (MPP) that entirely depends on the ambient meteorological conditions (i.e. solar irradiance and temperature). Hence, to continuously extract and deliver the maximum possible power from the PV system, under given meteorological conditions, the maximum power point tracking (MPPT) control strategy needs to be formulated that continuously operates the PV system at its MPP. To achieve this goal, a hybrid nonlinear, very fast and efficient MPPT control strategy, based on the robust integral backstepping (RIB) control, is formulated in this research article. The simulation testbed comprises a standalone PV array, a non-inverting buck-boost (NIBB) DC-DC power converter, a purely resistive and a dynamic load (sound system). The proposed MPPT control scheme consists of two loops, where the first loop generates the real-time offline reference peak power voltage through an adaptive neuro-fuzzy inference system (ANFIS) network, which is then utilized in the second loop as a set-point value for generating a control signal and then forcing the PV system to be operated at this set-point by continuously adjusting the duty ratio of the power converter. This control strategy exhibits no overshoot, fast convergence, good transient response, fast rising and settling times and minimum output tracking error. The MATLAB/Simulink platform is used to test the performance of the proposed MPPT strategy against varying meteorological conditions, plant current and voltage faults and plant parametric uncertainties. To validate the superiority of the proposed control strategy, a comparative analysis of the proposed control strategy is presented with the nonlinear backstepping (B), integral backstepping controller (IB) and conventional PID and P&O based MPPT controllers.

## 1 Introduction

Recently, worldwide energy demand has increased exponentially. More than 70% of the globally generated electricity is supplied by the fossil fuels (namely natural gas, coal and petroleum) [1]. Unfortunately, the available supply of these resources is shrinking abruptly, resulting in a challenging future for the energy production. Furthermore, the fossil fuels based power generation gives rise to greenhouse effect and global warming. In order to decrease the dependence on the fossil fuels for energy production in the world, it is essential to exploit renewable energy sources, including solar energy, wind energy, tidal energy, geothermal energy etc. Among these aforementioned energy resources, photovoltaic (PV) based energy production is a good choice owing to the fact that it is universally available, environment friendly, free of charge and has less operational and maintenance costs [2]. The need for PV based energy generation has increased both for grid-connected and standalone systems. However, solar panels have a nonlinear electrical characteristics (*I* − *V* and *P* − *V*), with a unique maximum power point (MPP), under uniform solar irradiance and temperature [3]. But, under the partial shading condition (PSC), the PV modules belonging to the same string experience different or nonuniform solar irradiance. Consequently, the solar array *P* − *V* curve deviates from its standard form and exhibits multiple local MPPs (LMPPs) [4, 5]. This condition may be caused by the movement of clouds or some obstacle (e.g. nearby trees or buildings, long-lasting dust etc.) causing a shade or shadow over the solar array. The PSC hinders an efficient MPPT operation. When a local instead of the global MPP (GMPP) is tracked, the result is the energy loss that can be significant (up to 70%), leading to the degradation of the overall system performance and efficiency. So, to uninterruptedly extract and deliver the maximum possible power from the PV system, it needs to be operated at its MPP, despite variations in the meteorological conditions.

The key objective of this research article is to carry out the optimum operation of the PV system based on the maximum power point tracking (MPPT) control strategy under uniform solar irradiance and temperature within a specific time interval. In the literature [6], numerous techniques on MPPT have been developed such as offline or indirect, online or direct and hybrid techniques.

Offline MPPT strategies, such as short circuit current (SCC) strategy [7, 8], open circuit voltage (OCV) strategy [9, 10], basically require some PV values to produce the control signal essential for operating the PV system at its MPP. Since, these techniques are simple and inexpensive, therefore, they have been frequently employed in the PV systems. However, the disadvantage of these MPPT control strategies is that they generally fail under varying meteorological conditions and are incapable of operating under PSC. Because, they are restricted to local search for the MPP, and can identify only a single MPP, but not the GMPP with the highest power output [11, 12].

Several MPPT regarding articles have been reported to have focused on the online techniques, such as extremely seeking control (ESC) method [13–15], perturb and observe (P&O) strategy [16–18] and incremental conductance (IC) method [19, 20]. These control schemes usually use PV current and voltage in real-time to achieve the MPP. The major drawback of these methods is oscillations around the MPP [21].

Hybrid MPPT control strategies [22–24] are basically the combination of both online and offline control strategies. For tracking the MPP, these techniques use two loops. In the first loop, an offline strategy is employed to estimate the real-time reference peak power voltage (*V*_*MPP*_) for the PV system. In the second loop, the estimated reference peak power voltage is used as a set-point for generating the control signal, *u*, and then forcing the PV system to be operated at this set-point by continuously adjusting the duty ratio, *d*, of the power electronic interface (power converter).

Typically, for a PV system, MPPT control strategy is a highly nonlinear control problem. Several MPPT based nonlinear control strategies have been reported in the literature for both grid-connected and stand-alone PV systems [25, 26]. In [27, 28], two loops hybrid nonlinear backstepping and integral backstepping MPPT controllers, respectively, have been proposed for a PV system with a purely resistive load. In the first loop, regression plane has been used to estimate the real-time offline MPP for the PV system. While in the second loop, nonlinear backstepping and integral backstepping based MPPT controllers have been formulated to force the PV system to be operated at their estimated MPPs. Both the control strategies have been found very faithful in achieving the MPPT either under varying temperature alone, or varying irradiance alone. Their performances have been found to be superior to the traditional P&O based MPPT technique. However, a substantial steady-state error and overshoot have been observed in the backstepping (B) and integral backstepping (IB) techniques, respectively. Also, the robustness of these techniques have not been tested for certain plant parametric uncertainties, plant voltage and current faults and dynamic load. Moreover, these techniques need to be further evaluated under simultaneous variation of temperature and solar irradiance.

The backstepping is a nonlinear recursive control design technique. The principal idea behind its application is the stabilization of the virtual control state [29, 30]. It is based on designing an MPPT controller recursively by choosing some of the system state variables as the virtual controllers, and then designing intermediate control laws for each of the selected virtual controller. This approach is well-suitable for boundary control problems. While the control is acting only from the boundary, its main feature is the capability of canceling out all the destabilizing effects (i.e. forces or terms) appearing throughout the domain. Its attractive features include: fast dynamic response, robustness to system parametric uncertainties, good performance against unmodeled system dynamics and external disturbance rejection [31, 32].

To mitigate the stated problems of the recently proposed backstepping and integral backstepping techniques [27, 28], a hybrid nonlinear robust integral backstepping (RIB) based MPPT control strategy is formulated in this research work. The proposed technique is tested on a PV system comprising a standalone PV array, a non-inverting buck-boost (NIBB) DC-DC power converter, a resistive load (comprising DC lighting) and a dynamic load (comprising sound system used in military parade grounds, large religious gatherings and holy worship places). The proposed control strategy contains two loops. The first loop estimates the real-time offline MPP, *V*_*MPP*_, through an adaptive neuro-fuzzy inference system (ANFIS). The second loop uses the estimated value of the MPP as a set-point for the robust integral backstepping strategy to generate the control signal, *u*, and then forcing the PV system to be operated at this set-point by continuously adjusting the duty ratio, *d*, of the NIBB DC-DC power electronic converter.

### 1.1 Significant contributions

The major contributions made by this research article are as follows:

1. A nonlinear hybrid RIB based MPPT scheme is formulated for a standalone PV system connected to a dynamic load, using ANFIS for real-time offline estimation of the MPP during the PV system operation.
2. The stability of the formulated MPPT scheme is guaranteed through the Lyapunov stability theory and MPPT is ensured under varying meteorological conditions subject to certain plant voltage and current faults and plant parametric uncertainties.
3. The proposed control scheme has been figured out in such a way that it should be simple to understand and easy to implement.
4. Through RIB controller, the MPPT is guaranteed with a superior performance to the benchmarks backstepping (B), integral backstepping (IB) and conventional PID and P&O based MPPT techniques.

The present research article is organized in the following manner: The reference peak power voltage estimation is covered in Section 2. The mathematical modeling of the overall PV system comprising the PV array, DC-DC converter and load is discussed in Section 3. Section 4 is about the proposed RIB based MPPT control strategy design. Performance validation and superiority of the proposed control scheme is validated through Matlab simulations in Section 5. Finally, Section 6 presents concluding remarks and future research recommendations.

## 2 Reference peak power voltage estimation through Adaptive Neuro-Fuzzy Inference System (ANFIS)

**Fig 1:**
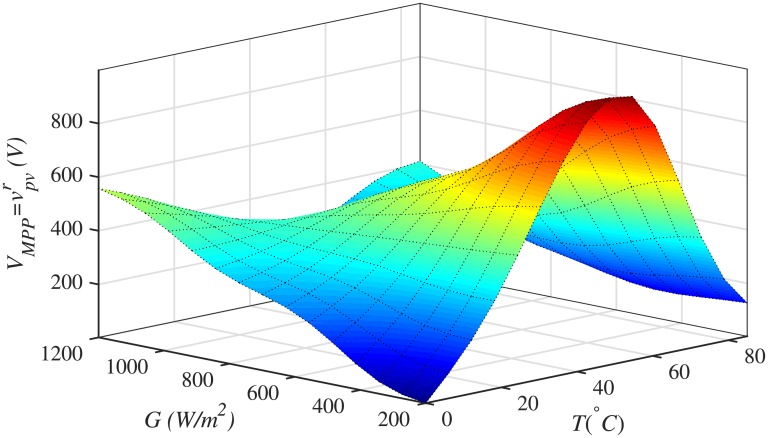
3D-surface for ANFIS.

In this work, ANFIS based on Takagi–Sugeno–Kang (TSK) is used for generating the reference peak power voltage, *V*_*MPP*_ or $v_{pv}^{r}$, of the PV array. The ANFIS network has temperature, *T*(°*C*), and irradiance, *G* (*W*/*m*^2^), as two input variables and *V*_*MPP*_ as an output variable. The input layer is the fuzzification layer, having three Gaussian membership functions (GMFs) for each input variable. The output layer consists of a linear equation for each rule. The 3D–surface, illustrated in Fig 1, represents the estimated *V*_*MPP*_ of the PV array through the ANFIS, under varying irradiance and temperature.

For ANFIS based *V*_*MPP*_ estimation, the *V*_*MPP*_ related input–output data is recorded by entering the user-defined PV array specifications, expressed in Table 1, in Matlab/Simulink. During this procedure, the temperature is perturbed from 20°*C* to 85°*C* in uniform steps of 1°*C*. On the other hand, the solar irradiance is perturbed from 600 *W*/*m*^2^ to 1, 000 *W*/*m*^2^, in uniform steps of 1 *W*/*m*^2^. As a result, about 26,500 input–output data points are recorded. This data set is then used to obtain an ANFIS based trained model.

**Table 1:** Overall parameters of the PV system.

Type	Name of Parameters	Symbols	Magnitude
**PV Array**	Maximum power per PV module	*P*_*max*_	1555 *W*
	Number of Cells per module	*N*_*s*_	72
	Number of module per PV array	−	16
	Module voltage at maximum power	*V*_*MPP*_	102.6 *V*
	Module open circuit voltage	*V*_*oc*_	165.8 *V*
	Module short circuit current	*I*_*sc*_	17.56 *A*
	Module current at maximum power	*I*_*MPP*_	151.16 *A*
**Converter**	Input Capacitor	*C*_1_	1 × 10^−3^ *F*
	Inductor	*L*	20 × 10^−3^ *H*
	Output capacitor	*C*_2_	48 × 10^−6^ *F*
	Load resistance	*R*_*L*_	50 *Ω*
	IGBT switching frequency	*f*_*s*_	5000 *Hz*
**Sound System**	Back emf constant	*K*_*b*_	15 *V*/(*m*/*s*)
	Speaker resistance	*R*_*s*_	10 *Ω*
	Speaker inductance	*L*_*s*_	10^−3^ H
	Proportional constant	*K*_*f*_	15 *N*/*A*
	Spring constant	*k*	5 × 10^5^ *N*/*m*
	Spring and diaphragm mass	*m*	0.001 *kg*
**Controller**	Constant	*k*_1_	60
	Constant	*k*_2_	9000
	Constant	*k*_3_	2000
	Constant	*k*_4_	10
	Constant	*λ*	29

**Fig 2:**
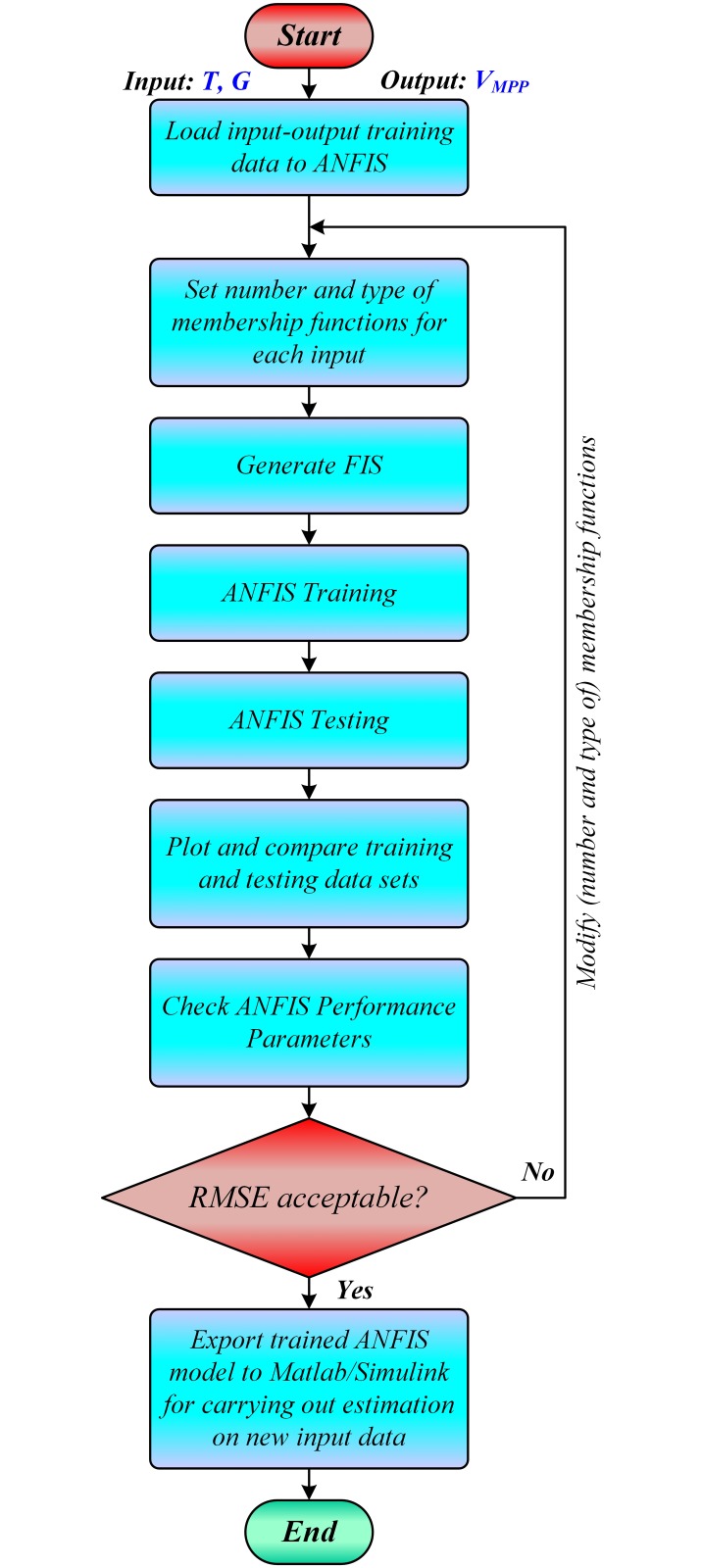
Computational flowchart for ANFIS based *V*_*MPP*_ learning.

The flowchart for working of ANFIS based *V*_*MPP*_ estimation is illustrated in Fig 2. The trained ANFIS based model is then exported to Simulink for estimating the real-time offline *V*_*MPP*_ of the PV array during simulation, for any combination of input temperature and irradiance levels, which is then tracked by the MPPT controller.

## 3 Overall PV system mathematical modeling

**Fig 3:**
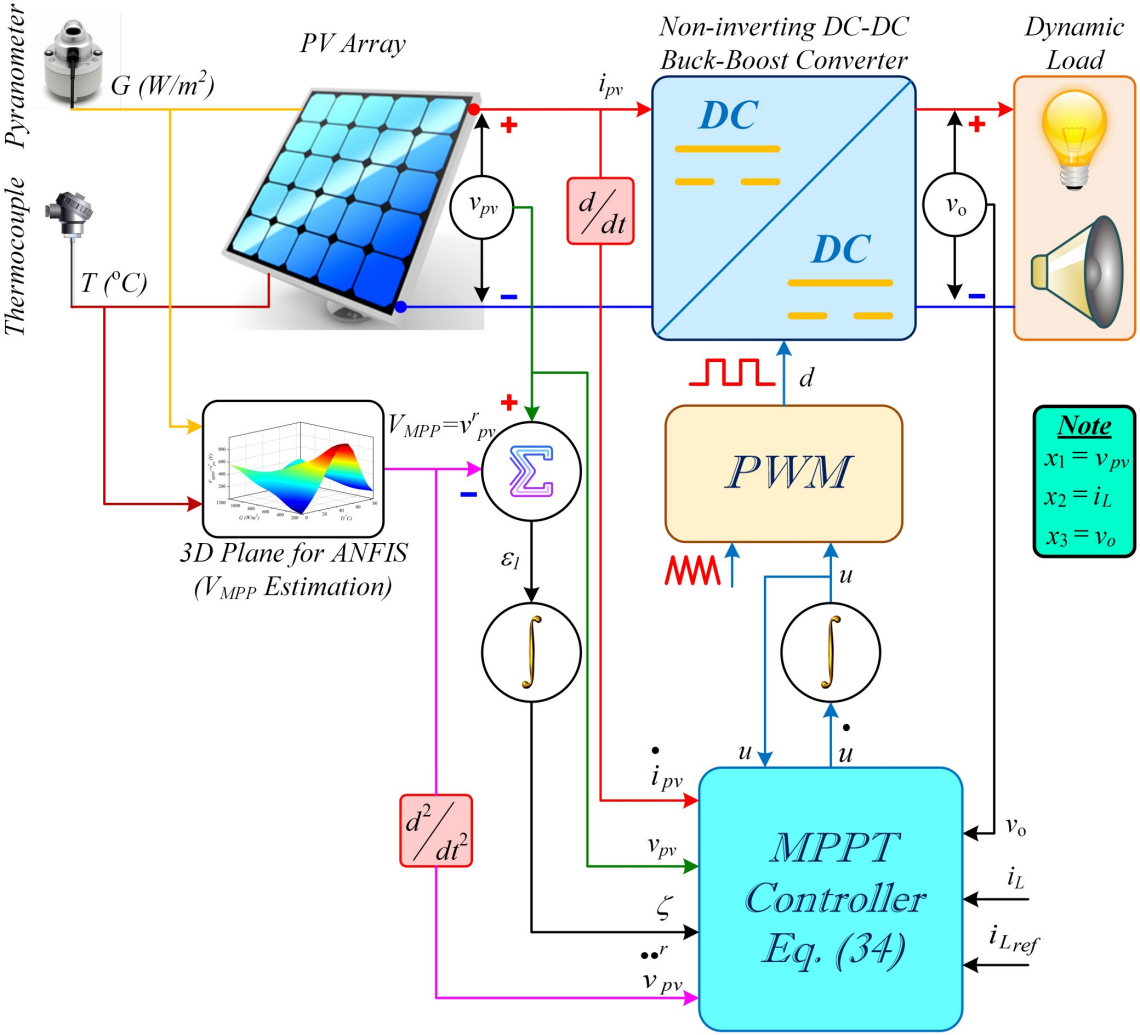
Block diagram of the overall proposed control system.

The block diagram of the overall proposed control system used in this study is depicted in Fig 3 that comprises a standalone PV array, a NIBB DC-DC power converter, a resistive load (comprising DC lighting), a dynamic load (comprising sound system used in military parade grounds, large religious gatherings and holy worship places) and an RIB based MPPT controller.

### 3.1 Standalone PV array mathematical modeling

**Fig 4:**
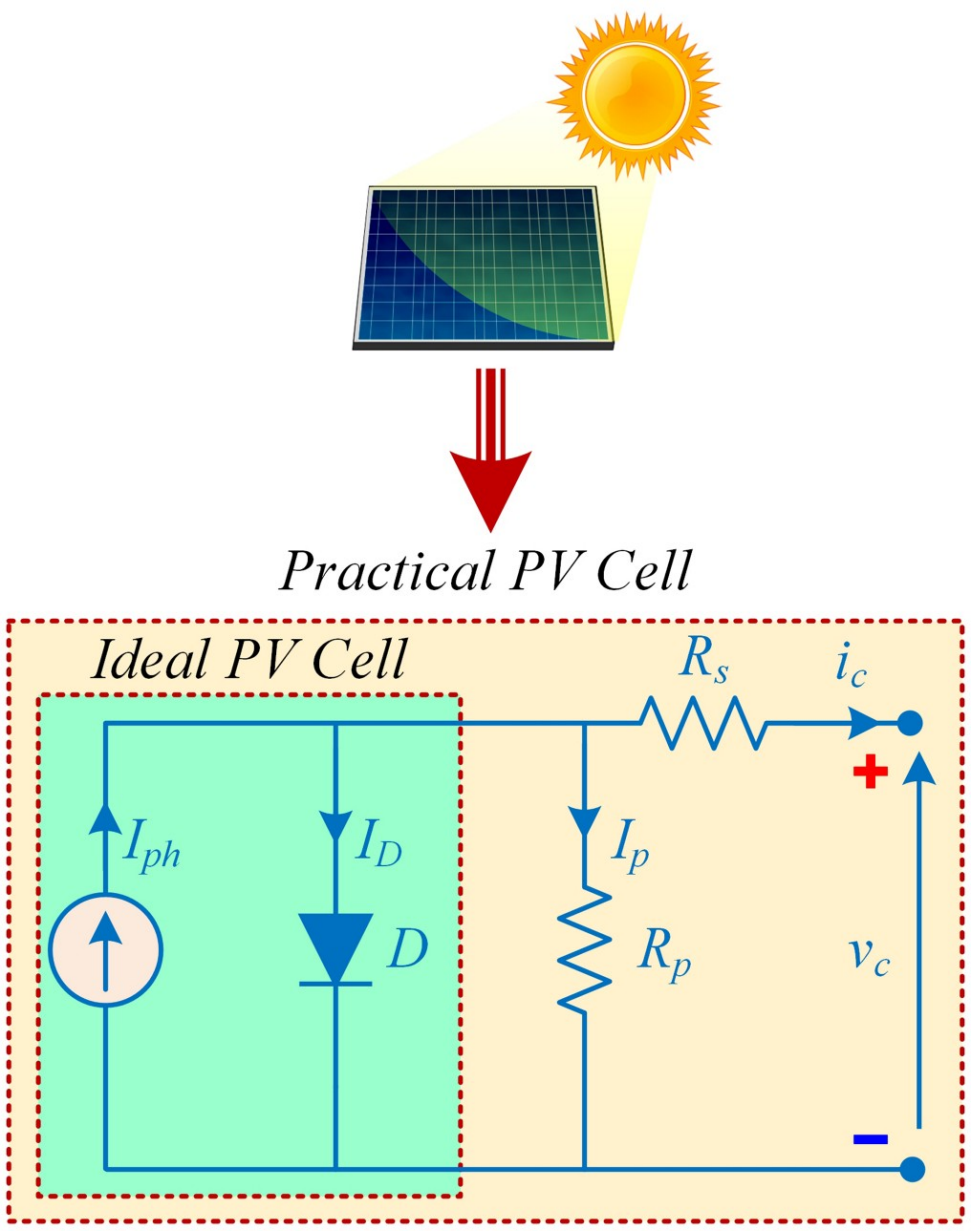
Equivalent single-diode circuit of a PV cell.

The PV cell contains a PN–junction just like an ordinary diode that generates electricity (DC) using photons. In order to get a better physical insight into a PV cell operation and its characteristics, equivalent circuit models are used. Depending on their complexity and accuracy, a PV cell can be represented by several different equivalent circuit models, such as: single-diode model (SDM), two-diode model (TwDM) and three-diode model (ThDM) [33]. The SDM is the simplest equivalent circuit model of a PV cell having reasonable accuracy, as illustrated in Fig 4. A practical model of PV cell, based on SDM, consists of a series resistance, *R*_*s*_, a shunt resistance, *R*_*p*_, a light-dependent current source, *I*_*ph*_, and an anti-parallel diode, *D*. Generally, *R*_*s*_ ≪*R*_*p*_, where *R*_*s*_ exists due to the metallic leads resistances, while *R*_*p*_ due to the leakage current of the PN–junction. Furthermore, *I*_*D*_, *I*_*p*_, *i*_*c*_ and *v*_*c*_ are the diode current, current through the shunt-resistance, cell output current and cell output voltage, respectively.

Mathematically, the PV cell output current, *i*_*c*_, can be obtained by applying Kirchhoff’s current law at the junction in Fig 4, as follows:

$$\begin{array}{c} i_{c}=I_{ph}-\underset{I_{D}}{\underset{︸}{I_{o}[e^{\frac{q}{AkT}(v_{c}+i_{c}R_{s})}-1]}}-\underset{I_{p}}{\underset{︸}{\frac{v_{c}+i_{c}R_{s}}{R_{p}}}} \end{array}$$ (1)

In Eq (1), *I*_*D*_ denotes the Shockley diode equation, *I*_*o*_ represents the diode leakage (or reverse saturation) current, *q* equals the electron charge (1.6 × 10^−19^ *C*), *k* is the Boltzmann constant (1.38 × 10^−23^ *J*/*K*), *T* represents the PN–junction temperature (*K*) and *A* denotes the diode ideality factor (or constant), where usually: 1 ≤ *A* ≤ 1.50.

For practical use, many PV cells are joined in series and parallel combination to obtain higher voltages and currents, respectively. Let, *N*_*p*_ and *N*_*s*_ represent the number of parallel-connected PV modules and series-connected PV cells, respectively. Then, the mathematical equation between the PV array output current, *i*_*pv*_, and output voltage, *v*_*pv*_, can be written as follows [34]:

$$\begin{array}{c} \begin{array}{cc} & i_{pv}=N_{p}I_{ph}-N_{p}I_{o}[e^{\frac{q}{AkT}(\frac{v_{pv}}{N_{s}}+\frac{i_{pv}R_{s}}{N_{p}})}-1]-\frac{N_{p}}{R_{p}}(\frac{v_{pv}}{N_{s}}+\frac{i_{pv}R_{s}}{N_{p}}) \end{array} \end{array}$$ (2)

**Fig 5:**
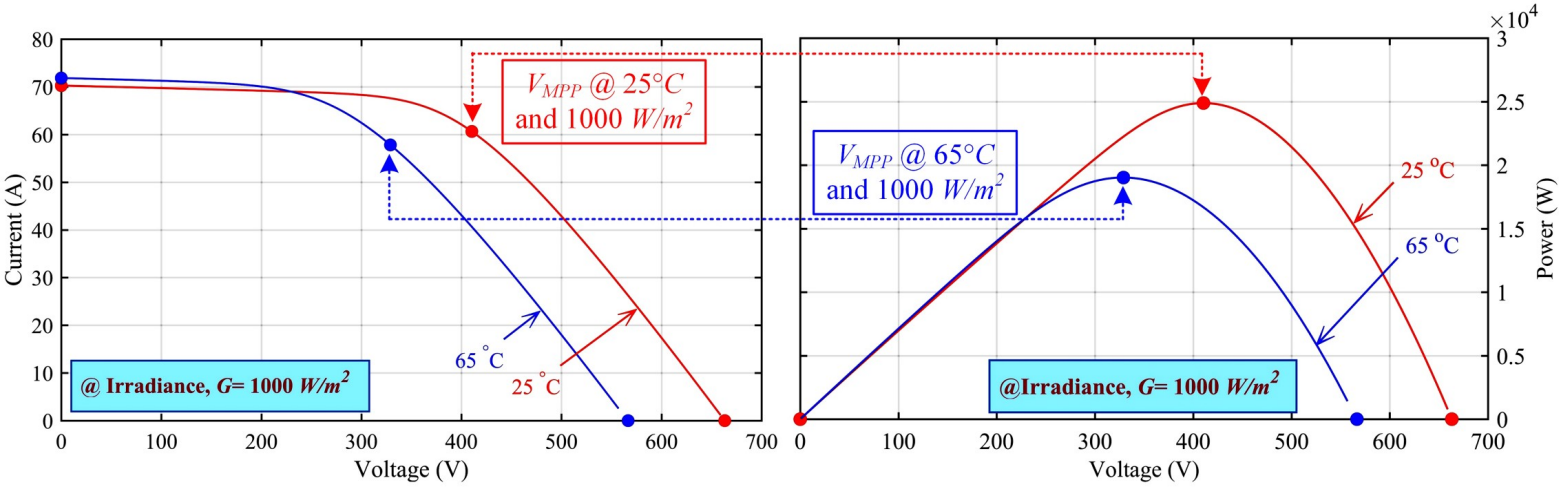
Electrical characteristics of the PV array.

A user-define standalone PV array has been considered in this work. It consists of 16 PV modules, each of 1, 555 *W*, where 4 PV modules are connected in series in each string and then 4 such strings are connected in parallel to constitute the complete PV array with a total power output of 24, 880 *W*. Table 1 describes different parameters of the PV array, under standard test conditions (STC), i.e. 25°*C* and 1, 000 *W*/*m*^2^. Furthermore, the PV array electrical characteristics (*I* − *V* and *P* − *V*), are illustrated in Fig 5.

### 3.2 Sound system (dynamic load) mathematical modeling

The main component of the sound system is as a speaker. It belongs to the class of electromechanical devices known as electro-acoustic. It is a dynamic load that is analogous to a permanent magnet DC (PMDC) motor. Its working principle is as follows: generally a stereo (or radio amplifier) produces a current in a coil that is attached to a diaphragm in the speaker cone. This causes the coil and diaphragm to move relative to the permanent magnet (PM). The motion of the diaphragm generates air pressure waves or sound [35].

**Fig 6:**
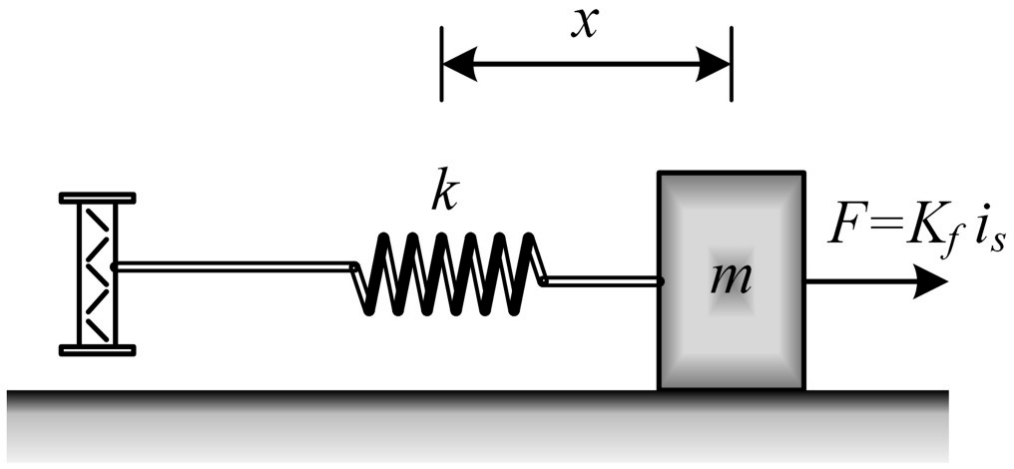
Mechanical subsystem of speaker.

The simplified model of the mechanical subsystem of the speaker is illustrated in Fig 6. Applying Newton’s law of motion to the mechanical subsystem of the speaker, it yields:

$$\begin{array}{c} m\frac{d^{2}x}{dt^{2}}=\underset{F}{\underset{︸}{K_{f}i_{s}}}-kx \end{array}$$ (3)

where *m* represents the combined mass of the speaker coil and diaphragm (*kg*), *x* denotes the diaphragm displacement, $\frac{d^{2}x}{dt^{2}}=a$ represents the diaphragm acceleration, *K*_*f*_ indicates the magnetic force constant (*N*/*A*), *i*_*s*_ represents the speaker current (*A*), *F* denotes the magnetic force (*N*) and *k* represents the spring constant (*N*/*m*).

**Fig 7:**
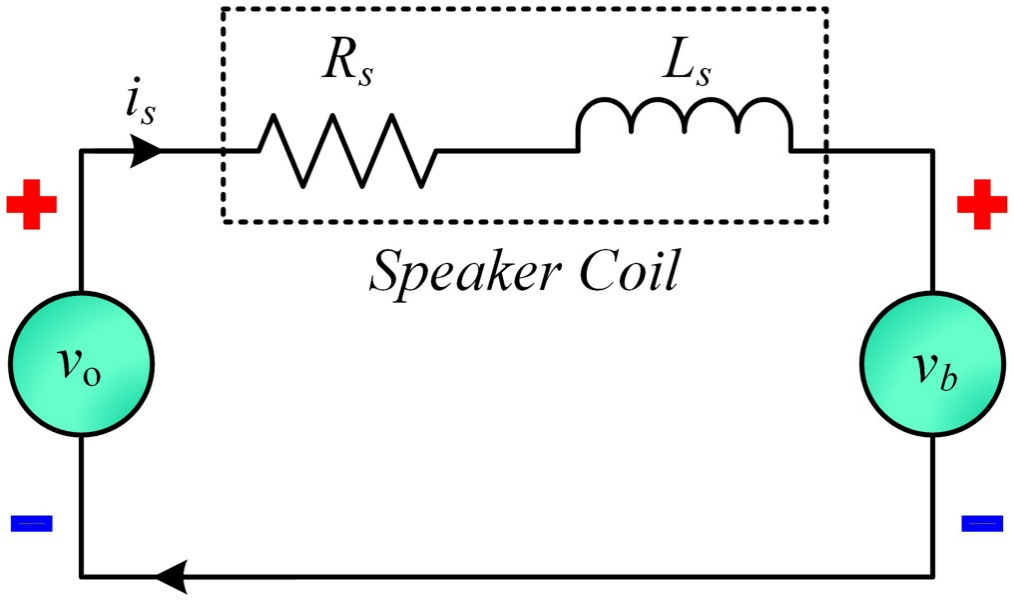
Electrical subsystem of speaker.

**Fig 8:**
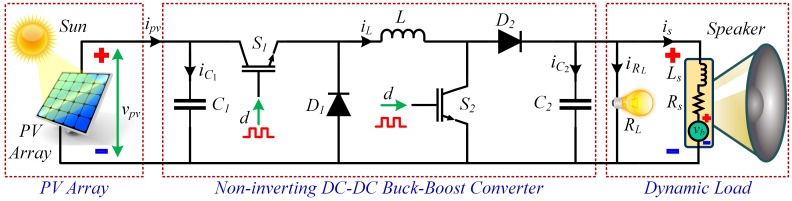
Equivalent circuit diagram of the NIBB DC-DC converter with source (standalone PV array) and dynamic load.

Similarly, the simplified model of the electrical subsystem of the speaker is illustrated in Figs 7 and 8. Applying Kirchhoff’s voltage law to the electrical subsystem of the speaker, it yields:

$$\begin{array}{c} v_{o}=L_{s}\frac{di_{s}}{dt}+R_{s}i_{s}+\underset{v_{b}}{\underset{︸}{K_{b}\frac{dx}{dt}}} \end{array}$$ (4)

where *v*_*o*_ is the applied voltage (*V*), *R*_*s*_ and *L*_*s*_ are the resistance (*Ω*) and inductance (*H*) of the speaker coil, respectively, *v*_*b*_ is the back emf (*V*), *K*_*b*_ is the back emf constant (*V*/(*m*/*s*)) and $\frac{dx}{dt}=\dot{x}=\omega$ is the velocity of the diaphragm.

Eqs (3) and (4) constitute the dynamic model of the speaker. In state-space representation, the speaker dynamic model can be expressed as follows:

$$\begin{array}{c} \frac{dx}{dt}=\omega \\ \frac{dx^{2}}{dt^{2}}=\frac{d\omega }{dt}=\frac{1}{m}\left(K_{f}i_{s}-kx\right)\\ \frac{di_{s}}{dt}=\frac{1}{L_{s}}\left(v_{o}-K_{b}\omega -R_{s}i_{s}\right) \end{array}\}$$ (5)

### 3.3 Non-inverting DC-DC buck-boost converter state-space average modeling

DC-DC converter serves as a power electronic interface for delivering power from the PV array to the load. For this purpose, several well-known variants of the converter have been used, such as, Cuk converter, boost converter, buck converter or conventional buck-boost converter. However, all these stated converters are prone to high switching stresses, and therefore reduced efficiency. Moreover, the polarity of the output voltage is reversed with respect to the polarity of the input voltage, especially in case of the Cuk converter and conventional buck-boost converter. These problems are solved by employing a NIBB DC-DC converter [36]. Therefore, in this research work, a NIBB power converter is employed as a power electronic interface between the DC source (standalone PV array) and the dynamic load, as illustrated in Fig 8. It has two diodes (*D*_1_ and *D*_2_) and two IGBT based controllable switches (*S*_1_ and *S*_2_), an input and output capacitor (*C*_1_ and *C*_2_, respectively) and an inductor *L*. This converter is a cascaded combination of two other converters (i.e. a buck converter and a boost converter). Moreover, it is capable of being operated in three separate operating modes, that is, a buck mode (with *S*_1_: ON, while *S*_2_: OFF), a boost mode (with *S*_1_: OFF, while *S*_2_: ON) and a buck-boost mode (with both *S*_1_ and *S*_2_: ON, concurrently). In this research article, the NIBB converter is operated in the buck-boost mode.

Throughout this research work, it is assumed that the converter operates in the continuous conduction mode (CCM). Furthermore, there are two different switching modes of operation for the stated converter in the buck-boost mode. Mode 1: *S*_1_ and *S*_2_ are ON, while diodes *D*_1_ and *D*_2_ remain OFF. Mode 2: *D*_1_ and *D*_2_ are ON, while *S*_1_ and *S*_2_ remain OFF.

Generally, linearized state-space average modeling technique is used to study a converter behavior and performance in different operating modes. For operation in Mode 1 of the NIBB converter, the corresponding, state-space equations in compact vector-matrix form are given as follows [37]:

$$\begin{array}{c} \begin{array}{cc} & \left[\begin{array}{c} \frac{dv_{pv}}{dt}\\ \frac{di_{L}}{dt}\\ \frac{dv_{o}}{dt}\\ \frac{di_{s}}{dt}\\ \frac{d\omega }{dt}\\ \frac{dx}{dt} \end{array}]=[\begin{array}{cccccc} 0 & -\frac{1}{C_{1}} & 0 & 0 & 0 & 0\\ \frac{1}{L} & 0 & 0 & 0 & 0 & 0\\ 0 & 0 & -\frac{1}{R_{L}C_{2}} & -\frac{1}{C_{2}} & 0 & 0\\ 0 & 0 & \frac{1}{L_{s}} & -\frac{R_{s}}{L_{s}} & 0 & 0\\ 0 & 0 & 0 & \frac{K_{f}}{m} & 0 & -\frac{k}{m}\\ 0 & 0 & 0 & 0 & 1 & 0 \end{array}][\begin{array}{c} v_{pv}\\ i_{L}\\ v_{o}\\ i_{s}\\ \omega \\ x \end{array}]+[\begin{array}{c} \frac{i_{pv}}{C_{1}}\\ 0\\ 0\\ -\frac{v_{b}}{L_{s}}\\ 0\\ 0 \end{array}\right] \end{array} \end{array}$$ (6)

Similarly, for operation in Mode 2 of the NIBB converter, the corresponding state-space equations in compact vector-matrix form are given as follows:

$$\begin{array}{c} \begin{array}{cc} & \left[\begin{array}{c} \frac{dv_{pv}}{dt}\\ \frac{di_{L}}{dt}\\ \frac{dv_{o}}{dt}\\ \frac{di_{s}}{dt}\\ \frac{d\omega }{dt}\\ \frac{dx}{dt} \end{array}]=[\begin{array}{cccccc} 0 & 0 & 0 & 0 & 0 & 0\\ 0 & 0 & -\frac{1}{L} & 0 & 0 & 0\\ 0 & \frac{1}{C_{2}} & -\frac{1}{R_{L}C_{2}} & -\frac{1}{C_{2}} & 0 & 0\\ 0 & 0 & \frac{1}{L_{s}} & -\frac{R_{s}}{L_{s}} & 0 & 0\\ 0 & 0 & 0 & \frac{K_{f}}{m} & 0 & -\frac{k}{m}\\ 0 & 0 & 0 & 0 & 1 & 0 \end{array}][\begin{array}{c} v_{pv}\\ i_{L}\\ v_{o}\\ i_{s}\\ \omega \\ x \end{array}]+[\begin{array}{c} \frac{i_{pv}}{C_{1}}\\ 0\\ 0\\ -\frac{v_{b}}{L_{s}}\\ 0\\ 0 \end{array}\right] \end{array} \end{array}$$ (7)

Now, the overall average state-space model of the NIBB converter, based on inductor volt-second balance and capacitor charge-balance principles, over one switching period, *T*_*s*_, in compact vector-matrix form is given as follows:

$$\begin{array}{c} \begin{array}{cc} & \left[\begin{array}{c} \frac{d{\bar{v}}_{pv}}{dt}\\ \frac{d{\bar{i}}_{L}}{dt}\\ \frac{d{\bar{v}}_{o}}{dt}\\ \frac{d{\bar{i}}_{s}}{dt}\\ \frac{d\bar{\omega }}{dt}\\ \frac{d\bar{x}}{dt} \end{array}]=[\begin{array}{cccccc} 0 & -\frac{d}{C_{1}} & 0 & 0 & 0 & 0\\ \frac{d}{L} & 0 & \frac{d-1}{L} & 0 & 0 & 0\\ 0 & \frac{1-d}{C_{2}} & -\frac{1}{R_{L}C_{2}} & -\frac{1}{C_{2}} & 0 & 0\\ 0 & 0 & \frac{1}{L_{s}} & -\frac{R_{s}}{L_{s}} & 0 & 0\\ 0 & 0 & 0 & \frac{K_{f}}{m} & 0 & -\frac{k}{m}\\ 0 & 0 & 0 & 0 & 1 & 0 \end{array}][\begin{array}{c} {\bar{v}}_{pv}\\ {\bar{i}}_{L}\\ {\bar{v}}_{o}\\ {\bar{i}}_{s}\\ \bar{\omega }\\ \bar{x} \end{array}]+[\begin{array}{c} \frac{i_{pv}}{C_{1}}\\ 0\\ 0\\ -\frac{v_{b}}{L_{s}}\\ 0\\ 0 \end{array}\right] \end{array} \end{array}$$ (8)

where in Eq (8), *d* is the duty ratio, ${\bar{v}}_{pv}$, ${\bar{i}}_{L}$, ${\bar{v}}_{o}$, $\bar{i_{s}}$, $\bar{\omega }$ and $\bar{x}$ are the average values of *v*_*pv*_, *i*_*L*_, *v*_*o*_, *i*_*s*_, *ω* and *x*, respectively. All the significant parameters of the NIBB DC-DC converter are expressed in Table 1.

## 4 Robust integral backstepping MPPT controller design

In this section, a nonlinear hybrid two-loop RIB based MPPT controller is designed that regulates the PV array output voltage, *v*_*pv*_, for maximum power extraction. The schematic of the overall control scheme is depicted in Fig 3. The ANFIS block estimates the real-time offline reference peak power voltage, *V*_*MPP*_ or $v_{pv}^{r}$, for any combination of the input temperature, *T*, and irradiance, *G*. The MPPT controller block (RIB controller) utilizes the estimated value of *V*_*MPP*_ as a set-point to generate a control signal, *u*, for adjusting the duty ratio, *d*, of the converter switches (*S*_1_ and *S*_2_) and forces the PV array output voltage (or converter input voltage), *v*_*pv*_, to track the *V*_*MPP*_.

To proceed with the proposed control system design, first of all, some assumptions are made as follows:

$$\begin{array}{c} {\bar{v}}_{pv}={\bar{v}}_{C_{1}}=x_{1}\\ {\bar{i}}_{L}=x_{2}\\ {\bar{v}}_{o}={\bar{v}}_{C_{2}}=x_{3}\\ {\bar{i}}_{s}=x_{4}\\ \bar{\omega }=x_{5}\\ \bar{x}=x_{6}\\ d=u \end{array}\}$$ (9)

Now, a PV array output voltage error, *ε*_1_, is defined as follows:

$$\begin{array}{c} \varepsilon_{1}=v_{pv}-v_{pv}^{r}=x_{1}-x_{1ref} \end{array}$$ (10)

where *x*_1_ = *v*_*pv*_ and $x_{1ref}=v_{pv}^{r}$ are the PV array actual output voltage and reference (or desired) peak power voltage, respectively. Differentiating Eq (10) with respect to time, and substituting value of ${\dot{v}}_{pv}={\dot{x}}_{1}$ from Eq (8), it becomes as follows:

$$\begin{array}{c} {\dot{\varepsilon }}_{1}={\dot{x}}_{1}-{\dot{x}}_{1ref}=(\frac{i_{pv}}{C_{1}}-u\frac{x_{2}}{C_{1}}-{\dot{x}}_{1ref}) \end{array}$$ (11)

Since, the key design objective is to provide a robust MPPT performance with almost zero steady-state error, therefore, an integral action, *ζ*, is introduced into Eq (10), as follows:

$$\begin{array}{c} e_{1}=\varepsilon_{1}+\lambda \zeta \end{array}$$ (12)

where *λ* is a positive design constant. Moreover, *ζ* is defined as follows:

$$\begin{array}{c} \zeta =\int_{0}^{\tau }\varepsilon_{1}d\tau \end{array}$$ (13)

As, the goal is to converge the error *ε*_1_ asymptotically to the origin, O, (equilibrium point), for this purpose, defining a Lyapunov candidate function, *V*_1_. In order to ensure the asymptotic stability of the system, *V*_1_ must satisfy these three conditions, (i). *V*_1_ must be positive definite, (ii). *V*_1_ must be radially unbounded, and (iii). *V*_1_ must have a negative-definite time derivative.

The selected Lyapunov function candidate, *V*_1_, is defined as follows:

$$\begin{array}{c} V_{1}=\frac{1}{2}\varepsilon_{1}^{2}+\frac{\lambda }{2}\zeta^{2} \end{array}$$ (14)

Now, differentiating Eq (14) with respect to time and substituting values of ${\dot{\varepsilon }}_{1}$ from Eq (11), and $\dot{\zeta }$ from Eq (13), one comes up with:

$$\begin{array}{c} {\dot{V}}_{1}=\varepsilon_{1}{\dot{\varepsilon }}_{1}+\lambda \zeta \dot{\zeta }=\varepsilon_{1}(\frac{i_{pv}}{C_{1}}-u\frac{x_{2}}{C_{1}}-{\dot{x}}_{1ref}+\lambda \zeta \varepsilon_{1}) \end{array}$$ (15)

For the time derivative of the Lyapunov candidate function, ${\dot{V}}_{1}$, to be negative-definite, let

$$\begin{array}{c} (\frac{i_{pv}}{C_{1}}-u\frac{x_{2}}{C_{1}}-{\dot{x}}_{1ref}+\lambda \zeta )=-k_{1}\varepsilon_{1}-k_{2}\mathrm{sign}\left(\varepsilon_{1}\right) \end{array}$$ (16)

where *k*_1_ and *k*_2_ are positive designed constants.

Rearranging Eq (16) yields

$$\begin{array}{c} x_{2}=(\frac{i_{pv}}{C_{1}}-{\dot{x}}_{1ref}+\lambda \zeta +k_{1}\varepsilon_{1}+k_{2}\mathrm{sign}(\varepsilon_{1}))\frac{C_{1}}{u} \end{array}$$ (17)

Substituting Eq (16) into Eq (15), it takes the following form:

$$\begin{array}{c} {\dot{V}}_{1}=-k_{1}\varepsilon_{1}^{2}-k_{2}\varepsilon_{1}\mathrm{sign}\left(\varepsilon_{1}\right) \end{array}$$ (18)

Now, treating the inductor current, *i*_*L*_ = *x*_2_ = *x*_2*ref*_, in Eq (17) as a virtual control input that acts as a stabilization function (i.e. reference or desired current) for the actual inductor current, *i*_*L*_ = *x*_2_. Consequently, one has the following new reference:

$$\begin{array}{c} x_{2ref}=(\frac{i_{pv}}{C_{1}}-{\dot{x}}_{1ref}+\lambda \zeta +k_{1}\varepsilon_{1}+k_{2}\mathrm{sign}(\varepsilon_{1}))\frac{C_{1}}{u} \end{array}$$ (19)

Now, to track *x*_2_ to its set-point or reference, *x*_2*ref*_, another error, *ε*_2_, is defined as follows:

$$\begin{array}{c} \varepsilon_{2}=x_{2}-x_{2ref}\;\text{or}\;x_{2}=\varepsilon_{2}+x_{2ref} \end{array}$$ (20)

Substituting *x*_2_ from Eq (20) into Eq (11), it yields:

$$\begin{array}{c} {\dot{\varepsilon }}_{1}=\frac{i_{pv}}{C_{1}}-u\frac{x_{2ref}}{C_{1}}-u\frac{\varepsilon_{2}}{C_{1}}-{\dot{x}}_{1ref} \end{array}$$ (21)

Substituting *x*_2*ref*_ from Eq (19) into Eq (21), it yields:

$$\begin{array}{c} {\dot{\varepsilon }}_{1}=-k_{1}\varepsilon_{1}-k_{2}\mathrm{sign}\left(\varepsilon_{1}\right)-\lambda \zeta -u\frac{\varepsilon_{2}}{C_{1}} \end{array}$$ (22)

Substituting Eq (22) into Eq (15), it yields:

$$\begin{array}{c} {\dot{V}}_{1}=-k_{1}\varepsilon_{1}^{2}-k_{2}\varepsilon_{1}\mathrm{sign}(\varepsilon_{1})-u\frac{\varepsilon_{1}\varepsilon_{2}}{C_{1}} \end{array}$$ (23)

The inequality expressed above can also be written as:

$$\begin{array}{c} {\dot{V}}_{1}=-2k_{1}V_{1}-\sqrt{2}k_{2}\sqrt{V_{1}}-u\frac{\varepsilon_{1}\varepsilon_{2}}{C_{1}} \end{array}$$ (24)

The details about this differential inequality will be presented at the end of this section.

Taking the derivative of Eq (20), it yields

$$\begin{array}{c} {\dot{\varepsilon }}_{2}={\dot{x}}_{2}-{\dot{x}}_{2ref} \end{array}$$ (25)

Now, taking the derivative of Eq (19) w.r.t time, by applying the quotient rule of derivatives, it becomes as follows:

$$\begin{array}{c} \begin{array}{cc} & {\dot{x}}_{2ref}=\frac{1}{u^{2}}[(u)({\dot{i}}_{pv}-C_{1}{\ddot{x}}_{1ref}+\lambda C_{1}\dot{\zeta }+k_{1}C_{1}{\dot{\varepsilon }}_{1})\\ & -(i_{pv}-C_{1}{\dot{x}}_{1ref}+\lambda C_{1}\zeta +k_{1}C_{1}\varepsilon_{1}+k_{2}C_{1}\mathrm{sign}(\varepsilon_{1}))(\dot{u})] \end{array} \end{array}$$ (26)

Substituting the value of $\dot{\varepsilon_{1}}$ from Eq (22), *x*_2*ref*_ from Eq (19) and $\dot{\zeta }$ from Eq (13), it yields:

$$\begin{array}{c} \begin{array}{cc} & {\dot{x}}_{2ref}=\frac{1}{u}[{\dot{i}}_{pv}-C_{1}{\ddot{x}}_{1ref}+\lambda C_{1}\varepsilon_{1}-C_{1}k_{1}^{2}\varepsilon_{1}-C_{1}k_{1}k_{2}\mathrm{sign}\left(\varepsilon_{1}\right)-C_{1}k_{1}\lambda \zeta -k_{1}u\varepsilon_{2}]\\ & -\frac{x_{2ref}\dot{u}}{u} \end{array} \end{array}$$ (27)

Substituting the value of ${\dot{x}}_{2ref}$ from Eq (27) into Eq (25), ${\dot{\varepsilon }}_{2}$ becomes as follows:

$$\begin{array}{c} \begin{array}{cc} & {\dot{\varepsilon }}_{2}={\dot{x}}_{2}-\frac{1}{u}[{\dot{i}}_{pv}-C_{1}{\ddot{x}}_{1ref}+\lambda C_{1}\varepsilon_{1}-C_{1}k_{1}^{2}\varepsilon_{1}-C_{1}k_{1}k_{2}\mathrm{sign}\left(\varepsilon_{1}\right)-C_{1}k_{1}\lambda \zeta ]+k_{1}\varepsilon_{2}\\ & +\frac{x_{2ref}\dot{u}}{u} \end{array} \end{array}$$ (28)

Now, to ensure the asymptotic stability in the closed-loop and the convergence of both the error signals, *ε*_1_ and *ε*_2_, to the equilibrium point, a new composite Lyapunov candidate function, *V*_2_, is selected under the same assumptions as those made for *V*_1_, as follows:

$$\begin{array}{c} V_{2}=V_{1}+\frac{1}{2}\varepsilon_{2}^{2} \end{array}$$ (29)

Differentiating Eq (29) w.r.t time and substituting ${\dot{V}}_{1}$ from Eq (23), it becomes as follows:

$$\begin{array}{c} {\dot{V}}_{2}={\dot{V}}_{1}+\varepsilon_{2}{\dot{\varepsilon }}_{2}=-k_{1}\varepsilon_{1}^{2}-k_{2}\varepsilon_{1}\mathrm{sign}(\varepsilon_{1})+\varepsilon_{2}({\dot{\varepsilon }}_{2}-u\frac{\varepsilon_{1}}{C_{1}}) \end{array}$$ (30)

For the second Lyapunov candidate function, ${\dot{V}}_{2}$, to be negative-definite, let

$$\begin{array}{c} \left({\dot{\varepsilon }}_{2}-u\frac{\varepsilon_{1}}{C_{1}})=-k_{3}\varepsilon_{2}-k_{4}\mathrm{sign}(\varepsilon_{2}\right) \end{array}$$ (31)

where *k*_3_ and *k*_4_ are positive design constants.

Comparing Eqs (30) and (31), it yields:

$$\begin{array}{c} {\dot{V}}_{2}=-k_{1}\varepsilon_{1}^{2}-k_{2}\varepsilon_{1}\mathrm{sign}(\varepsilon_{1})-k_{3}\varepsilon_{2}^{2}-k_{4}\varepsilon_{2}\mathrm{sign}(\varepsilon_{2}) \end{array}$$ (32)

Now, substituting ${\dot{\varepsilon }}_{2}$ from Eq (28) and ${\dot{x}}_{2}$ from Eq (8) into Eq (31), it gives:

$$\begin{array}{c} \begin{array}{cc} & -k_{3}\varepsilon_{2}-k_{4}\mathrm{sign}(\varepsilon_{2})=\frac{ux_{1}}{L}+\frac{\left(u-1\right)x_{3}}{L}-\frac{1}{u}[{\dot{i}}_{pv}-C_{1}{\ddot{x}}_{1ref}+\lambda C_{1}\varepsilon_{1}-C_{1}k_{1}^{2}\varepsilon_{1}\\ & -C_{1}k_{1}k_{2}\mathrm{sign}\left(\varepsilon_{1}\right)-C_{1}k_{1}\lambda \zeta ]+k_{1}\varepsilon_{2}+\frac{x_{2ref}\dot{u}}{u}-u\frac{\varepsilon_{1}}{C_{1}} \end{array} \end{array}$$ (33)

Now, rearranging and solving Eq (33) for $\dot{u}$, the proposed complete RIB based MPPT control law becomes as follows:

$$\begin{array}{c} \begin{array}{cc} & \dot{u}=\frac{u}{x_{2ref}}[-k_{3}\varepsilon_{2}-k_{4}\mathrm{sign}(\varepsilon_{2})+u\frac{\varepsilon_{1}}{C_{1}}-k_{1}\varepsilon_{2}-\frac{C_{1}k_{1}\lambda \zeta }{u}]-\frac{u}{x_{2ref}}[\frac{ux_{1}}{L}+\frac{\left(u-1\right)x_{3}}{L}]\\ & +\frac{u}{x_{2ref}}[(\frac{1}{u})({\dot{i}}_{pv}-C_{1}{\ddot{x}}_{1ref}+\lambda C_{1}\varepsilon_{1}-C_{1}k_{1}^{2}\varepsilon_{1}-C_{1}k_{1}k_{2}\mathrm{sign}\left(\varepsilon_{1}\right))] \end{array} \end{array}$$ (34)

This choice of the dynamic control input, $\dot{u}$, reduces Eq (32) to the following differential inequality:

$$\begin{array}{c} {\dot{V}}_{2}\leq -2{\bar{k}}_{1}V_{2}-\sqrt{2}\;{\bar{k}}_{2}\sqrt{V_{2}} \end{array}$$ (35)

where ${\bar{k}}_{1}=min\left(k_{1},k_{3}\right)$ and ${\bar{k}}_{2}=min\left(k_{2},k_{4}\right)$. The differential equation Eq (35) looks very similar to the fast terminal attractor [38]. This confirms that *V*_2_ → 0 with finite settling time $t_{s_{2}}\leq \frac{1}{4{\bar{k}}_{1}}ln(\frac{2{\bar{k}}_{1}\sqrt{V_{2}}\left(0\right)+\sqrt{2}\;{\bar{k}}_{2}}{\sqrt{2}\;{\bar{k}}_{2}})$. In other words, *ε*_2_ = *x*_2_ − *x*_2*ref*_ → 0 in finite-time which confirms high precision in reference tracking as well as in errors and states regulation problems. As *ε*_2_ → 0 in finite-time, the last term in the differential equation Eq (24) vanishes. Consequently, a terminal attractor in terms of *V*_1_ is obtained which, once again, confirms the fast finite-time convergence of *ε*_1_ to zero. This convergence of *ε*_1_ is estimated to be $t_{s_{1}}\leq \frac{1}{4{\bar{k}}_{1}}ln(\frac{2{\bar{k}}_{1}\sqrt{V_{2}}\left(0\right)+\sqrt{2}\;{\bar{k}}_{2}}{\sqrt{2}\;{\bar{k}}_{2}})$. Hence, the proposed control law Eq (34), along with the virtual control law, Eq (19) regulates the respective error dynamics to zero in finite-time with high precision. Consequently, the reference tracking is achieved which will provide maximum power at each time instant.

Now, at this state, it is necessary to discuss the stability of the zero dynamics.

### 4.1 Stability of zero dynamics

Since, a two step integral backstepping based MPPT law is designed, therefore, the the following dynamic equations, expressed in Eq (8), are straight-a-way the internal dynamics of this proposed PV system:

$$\begin{array}{c} {\bar{\dot{x}}}_{3}=\frac{\left(1-u\right){\bar{x}}_{2}}{C_{2}}-\frac{{\bar{x}}_{3}}{R_{L}C_{2}}-\frac{{\bar{x}}_{4}}{C_{2}}\\ {\bar{\dot{x}}}_{4}=\frac{{\bar{x}}_{3}}{L_{s}}-\frac{R_{s}{\bar{x}}_{4}}{L_{s}}-\frac{v_{b}}{L_{s}}\\ {\bar{\dot{x}}}_{5}=\frac{K_{f}{\bar{x}}_{4}}{m}-\frac{k{\bar{x}}_{6}}{m}\\ {\bar{\dot{x}}}_{6}=\bar{\omega } \end{array}\}$$ (36)

According to the nonlinear control theory [39], the zero dynamics can be obtained by substituting the the applied control input, *u*, and the control driven states, *x*_1_ and *x*_2_, equal to zero into the internal dynamics, Eq (36). Thus, one has the following zero dynamics:

$$\begin{array}{c} \begin{array}{cc} & \left[\begin{array}{c} {\bar{\dot{x}}}_{3}\\ {\bar{\dot{x}}}_{4}\\ {\bar{\dot{x}}}_{5}\\ {\bar{\dot{x}}}_{6} \end{array}]=[\begin{array}{cccc} -\frac{1}{R_{L}C_{2}} & -\frac{1}{C_{2}} & 0 & 0\\ \frac{1}{L_{s}} & \frac{-R_{s}}{L_{s}} & 0 & 0\\ 0 & \frac{K_{f}}{m} & 0 & -\frac{k}{m}\\ 0 & 0 & 1 & 0 \end{array}][\begin{array}{c} {\bar{x}}_{3}\\ {\bar{x}}_{4}\\ {\bar{x}}_{5}\\ {\bar{x}}_{6} \end{array}]+[\begin{array}{c} 0\\ \frac{-v_{b}}{L_{s}}\\ 0\\ 0 \end{array}\right] \end{array} \end{array}$$ (37)

This is a linear time invariant (LTI) non-homogeneous system that can be represented, in general form, as follows:

$$\begin{array}{c} \dot{\bar{x}}=\bar{A}\bar{x}+\bar{B} \end{array}$$ (38)

where $\bar{x}={\left[x_{3},x_{4},x_{5},x_{6}\right]}^{T}$ represents zero dynamics state vector, $\bar{A}$ is the respective system distribution matrix, and $\bar{B}$ is a vector of time varying non-vanishing disturbances, which depend on the speaker back emf, *v*_*b*_. This system has general solution of the following form:

$$\begin{array}{c} \bar{x}\left(t\right)=e^{\bar{A}t}\bar{x}\left(0\right)+e^{\bar{A}t}\int_{t_{0}}^{t}e^{-r\bar{A}}\bar{B}dr \end{array}$$ (39)

Since, all the typical plant parameters are positive, therefore, the system Eq (37) of zero dynamics has two poles on the *jw* − *axis* i.e. at $\pm j-\frac{k}{m}$ and two poles in the left-half plan (LHP) at $-\frac{R_{s}R_{L}C_{2}+L_{s}}{2R_{L}L_{s}C_{2}}\pm \frac{1}{2}\sqrt{{\left(\frac{R_{s}R_{L}C_{2}+L_{s}}{2R_{L}L_{s}C_{2}}\right)}^{2}-8\frac{R_{L}}{R_{L}L_{s}C_{2}}}$. Note that, as long as the discriminant in the square root remains negative, it will give rise to conjugate poles with negative real parts. Thus, the exponential term $e^{\bar{A}t}$, which can be calculated via Caylay-Hamilton approach on the spectrum of the above defined LHP poles, has two oscillatory modes and two modes with decaying oscillations. Based on the information of the poles, the overall response of the zero dynamics will initially observe decaying oscillatory response to the vicinity of the origin, and then all the states will remain ultimately bounded. In other words, this also confirms that initially the zero dynamics will show a minimum phase nature, and as the effects of the exponential terms die out, the over all zero dynamics will stay bounded in a small neighborhood. This confirms the practical asymptotic convergence of the zero dynamics. Hence, the control will effectively track the reference in the presence of the practical asymptotic convergence of the internal dynamics.

## 5 Simulation results and discussion

**Fig 9:**
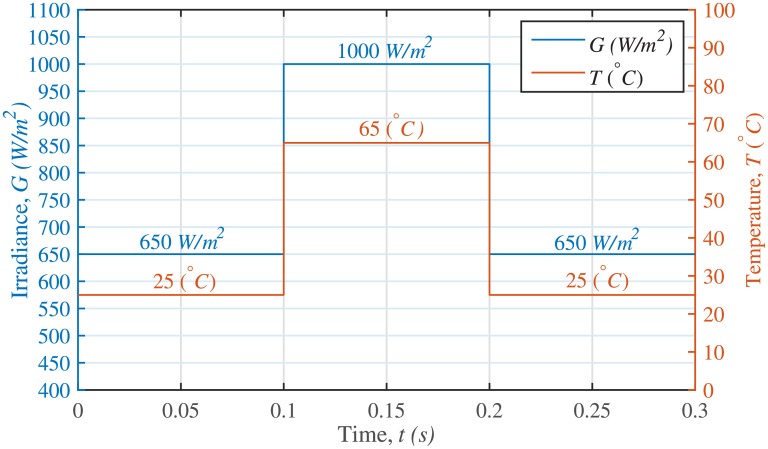
Irradiance and temperature profiles.

The Matlab/Simulink platform is used to test and validate the superiority of the proposed RIB based MPPT technique. The specifications of the MPPT controller, PV array, NIBB DC-DC power converter and sound system used in this research are given in Table 1. The irradiance and temperature profiles are shown in Fig 9. The proposed technique is evaluated and compared with the backstepping [27] and integral backstepping [28] based MPPT controllers under the following three different operating conditions:

**Case 1:** Performance test against varying meteorological conditions.
**Case 2:** Performance test against plant faults under varying meteorological conditions.
**Case 3:** Performance test against plant parametric uncertainties under varying meteorological conditions.

Finally, comparison with other conventional PID and P&O based MPPT techniques, under the stated three conditions, is also conducted.

### 5.4.1 Performance test against varying meteorological conditions

In this case study, the performance of the proposed MPPT control strategy is analyzed under varying meteorological conditions and then compared with the backstepping (B) and integral backstepping (IB) based MPPT control strategies.

**Fig 10:**
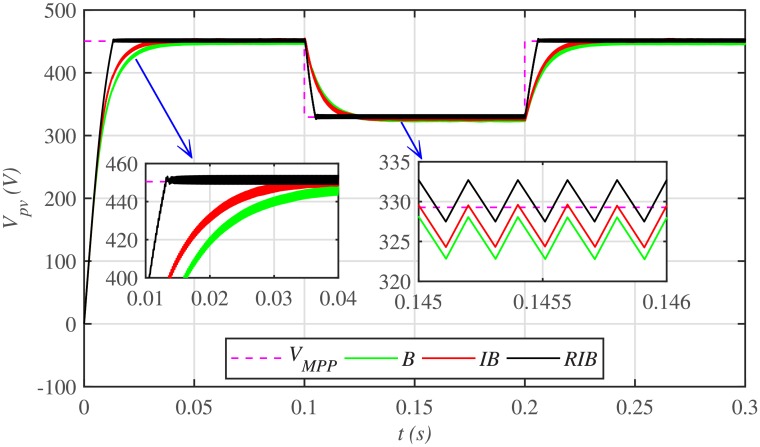
PV array comparative voltages under case 1.

**Fig 11:**
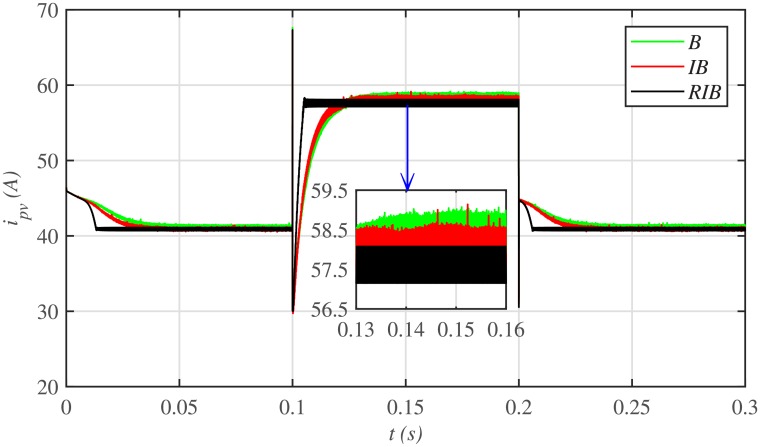
PV array comparative currents under case 1.

The comparative plot for the PV array output voltages, under case 1 is depicted in Fig 10. It can be seen from the zoomed-in view of Fig 10 that the proposed MPPT control strategy starts tracking the reference voltage (*V*_*MPP*_) in around 0.01 *s*, outperforming the other two MPPT techniques. This shows that the proposed control strategy has the lesser rising time, for achieving the MPPT, compared to the backstepping and integral backstepping techniques. Similarly, after abrupt change in meteorological conditions at 0.1 *s* and 0.2 *s*, the proposed technique performs very well by converging and settling earlier than the other two MPPT candidates. Throughout the simulation, the backstepping controller suffers from steady-state error, as shown in the zoomed-in view of Fig 10. Moreover, Fig 11 indicates the PV array output current. It is evident that the proposed control strategy extracts much smoother current from the PV array than its contenders.

**Fig 12:**
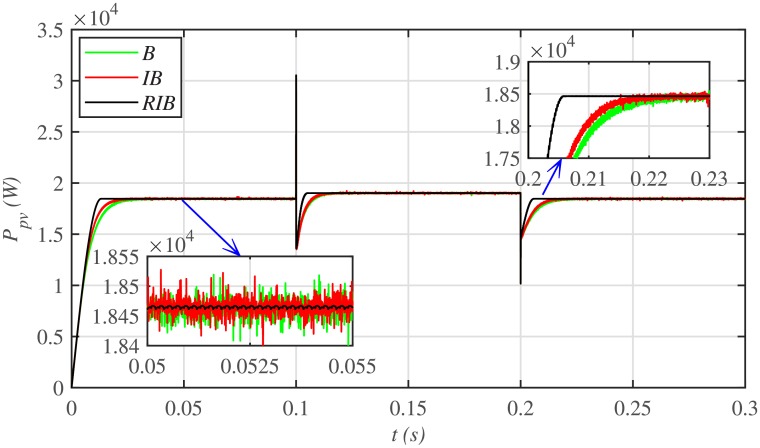
PV array comparative powers under case 1.

**Fig 13:**
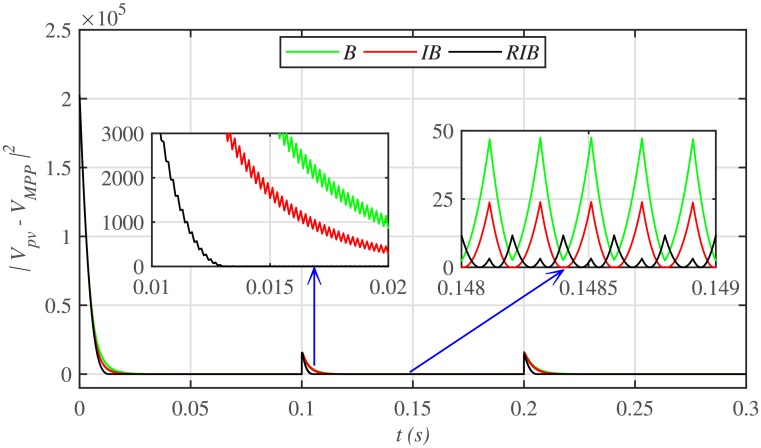
PV array output voltage error comparison under case 1.

Fig 12 depicts the comparative plot for the PV array output powers under case 1. Again, the proposed technique is robust and better than the other two MPPT techniques, in terms of lesser rising time, faster convergence, and offering almost negligible chattering, as shown in the zoomed-in view of Fig 12. Moreover, the proposed technique renders the minimum PV array output voltage tracking error (|*v*_*pv*_ − *V*_*MPP*_|), as illustrated in Fig 13, thus guaranteeing the accurate MPPT.

**Fig 14:**
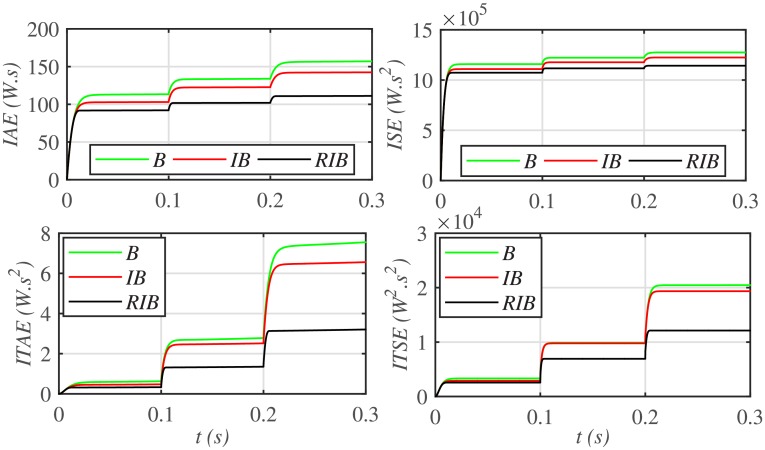
Performance indices comparison under case 1.

**Fig 15:**
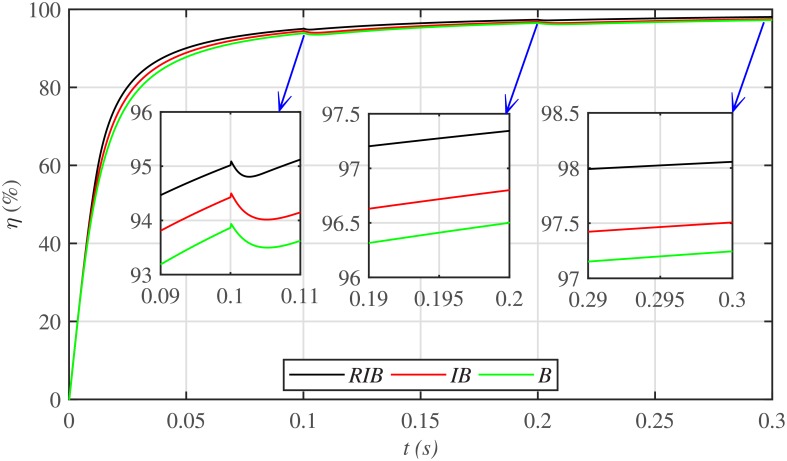
PV array efficiencies comparison under case 1.

The three MPPT candidates are also compared on the basis of the well-known performance indices (*ISE*, *ITSE*, *IAE* and *ITAE*), as depicted in Fig 14. It can be seen that the proposed paradigm renders the minimum accumulative error and the flattest error profile. It means that the proposed strategy is superior to both the backstepping and integral backstepping techniques. Furthermore, the proposed technique successfully delivers the maximum power from the PV array to the load with more than 98% efficiency, which is the maximum efficiency as compared to the other two techniques, as depicted in Fig 15.

### 5.4.2 Performance test against faults under varying meteorological conditions

During practical operation, the PV system is prone to certain faults that degrades its overall performance. This case study tests and compares the performance of the proposed control strategy with backstepping and integral backstepping strategies under case 2. For this purpose, a time varying sinusoidal fault *x*_3*f*_ = 30*usin*(*t*)/*C*_1_ is injected into the output capacitor voltage, *x*_3_, during the time interval 0.06 − 0.08 *s*, which resulted in Δ*x*_3_ = *x*_3_+ *x*_3*f*_. Similarly, another time varying sinusoidal fault *x*_2*f*_ = 0.5*usin*(*t*)/*C*_1_, is injected into the inductor current, *x*_2_, during the time interval 0.16 − 0.18 *s*, which resulted in Δ*x*_2_ = *x*_2_+ *x*_2*f*_.

**Fig 16:**
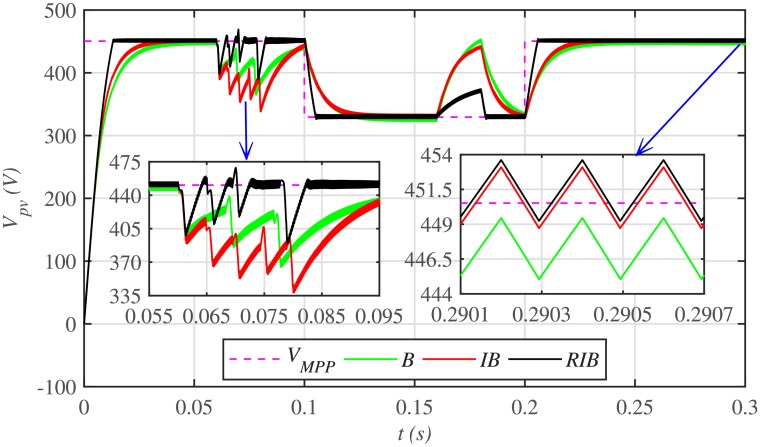
PV array comparative voltages under case 2.

**Fig 17:**
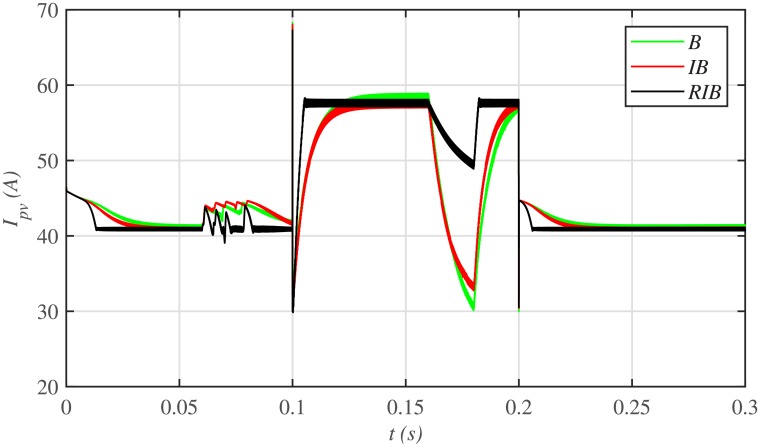
PV array comparative currents under case 2.

**Fig 18:**
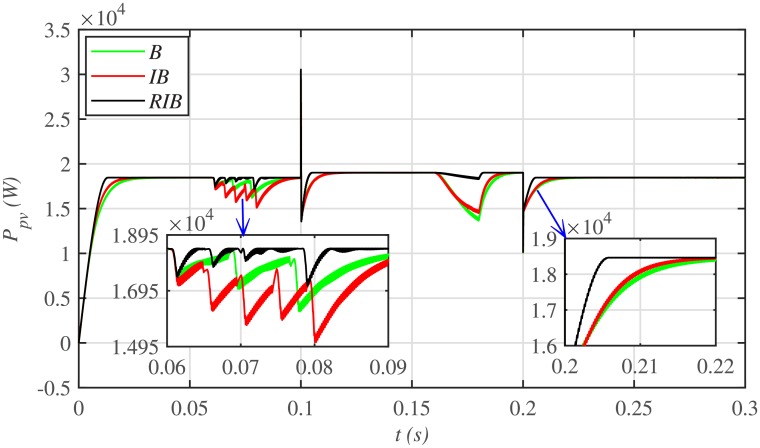
PV array comparative powers under case 2.

The comparative plot for the PV array output voltages under case 2 is shown in Fig 16. It is evident that all the MPPT controllers deviates from the *V*_*MMP*_, thereby losing tracking at the onset of faults in the plant. However, the proposed RIB control strategy deviates the minimum, thus offering more robustness against faults, as compared to the backstepping and integral backstepping strategy. Again, the backstepping controller suffers from steady-state error, as depicted in the zoomed-in view of Fig 16. Moreover, Fig 17 indicates the PV array output current. It is evident that the proposed control strategy extracts much smoother current from the PV array than its contenders. Similarly, the comparative plot for the PV array output powers under case 2 is illustrated in Fig 18. It can be seen that the proposed control scheme recovers and reaches the steady-state earlier than the other two MPPT candidates, once the faults are over.

**Fig 19:**
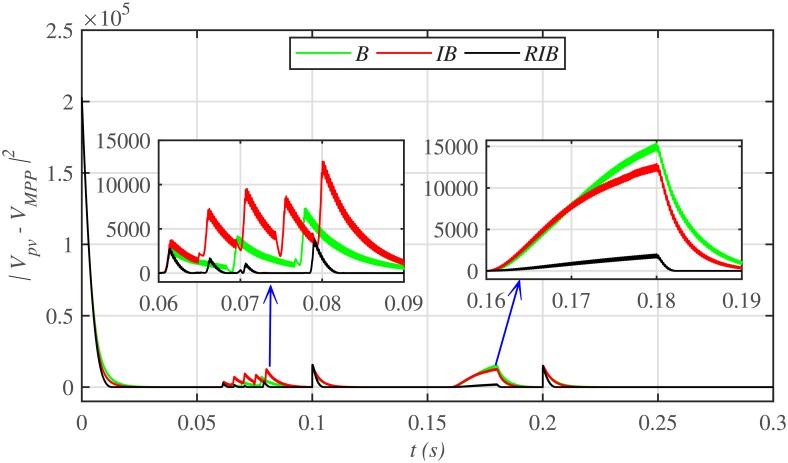
PV array output voltage error comparison under case 2.

**Fig 20:**
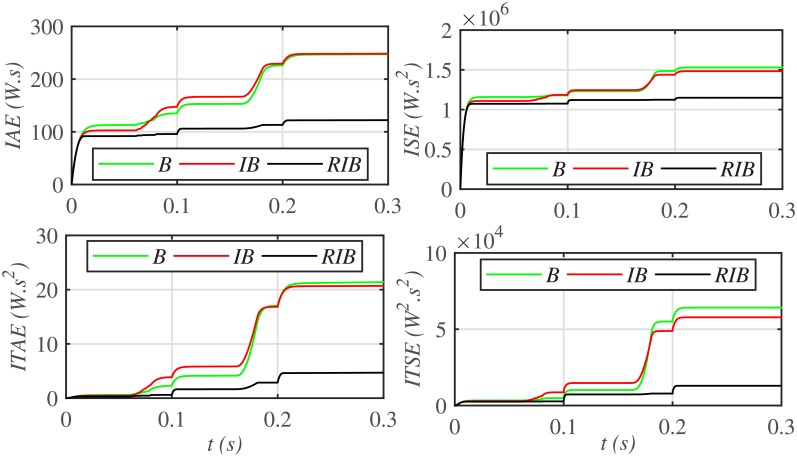
Performance indices comparison under case 2.

**Fig 21:**
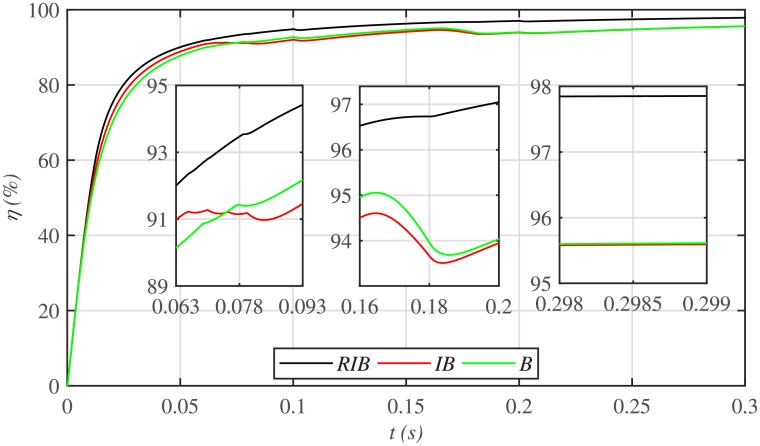
PV array efficiencies comparison under case 2.

Fig 19 depicts the PV array output voltage tracking error, whereas Fig 20 illustrates various performance indices under case 2. Again the proposed strategy achieves the MPPT with the minimum error, thus making it the best MPPT candidate under case 2. Similarly, Fig 21 compares the efficiencies of the three MPPT candidates, where it can be seen that the proposed strategy renders the highest efficiency of around 98%.

### 5.4.3 Performance test against parametric uncertainties under varying meteorological conditions

In this case study, the superiority of the proposed controller is tested and validated against plant parametric uncertainties and then compared with the backstepping and integral backstepping based MPPT controllers under case 3. This is carried out by introducing uncertainties into the converter inductor, *L*, and output capacitor, *C*_2_. Such that, from 0.06 − 0.08 *s*, *L* is increased by Δ*L* = 200 *mH*, which resulted in *L*_*new*_ = *L* + Δ*L*. Similarly, *C*_2_ is increased by Δ*C* = 0.48 *μF* from 0.16 − 0.18 *s*, which resulted in *C*_*new*_ = *C*_2_ + Δ*C*.

**Fig 22:**
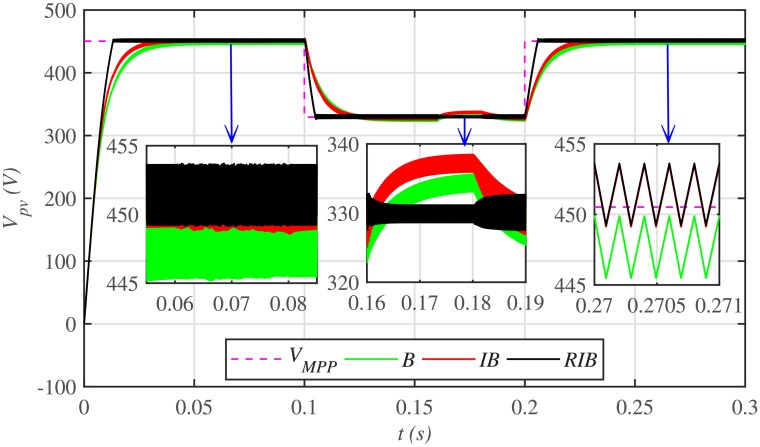
PV array output comparative voltages under case 3.

**Fig 23:**
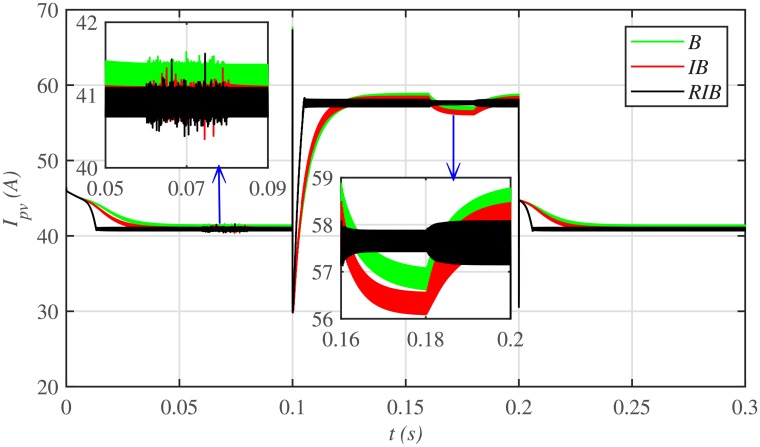
PV array comparative currents under case 3.

Fig 22 illustrates the comparative plot for the PV array output voltages under case 3. It can be observed from the zoomed-in view that both the capacitive and inductive uncertainties deviates the backstepping and integral backstepping control strategies from *V*_*MPP*_, however, the proposed RIB control strategy shows more robustness against plant uncertainties and remains unaffected. Moreover, Fig 23 illustrates the PV array output current. It is evident that the proposed control strategy extracts much smoother current from the PV array than its contenders.

**Fig 24:**
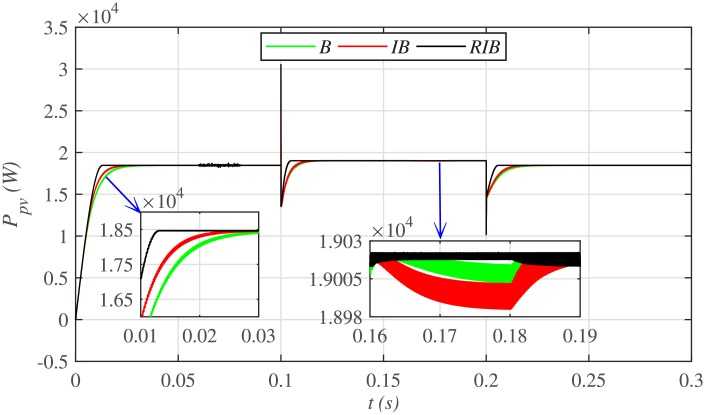
PV array comparative powers under case 3.

**Fig 25:**
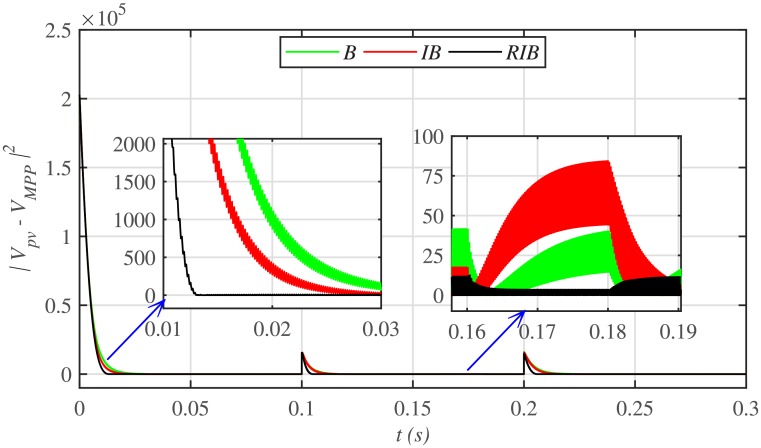
PV array output voltage tracking error under case 3.

**Fig 26:**
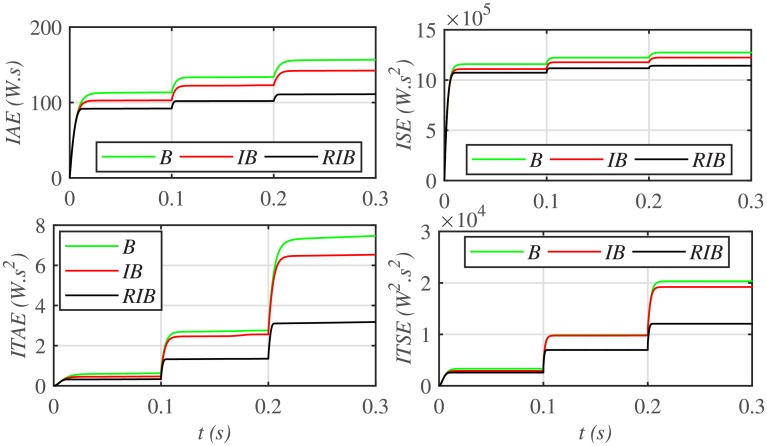
Performance indices comparison under case 3.

**Fig 27:**
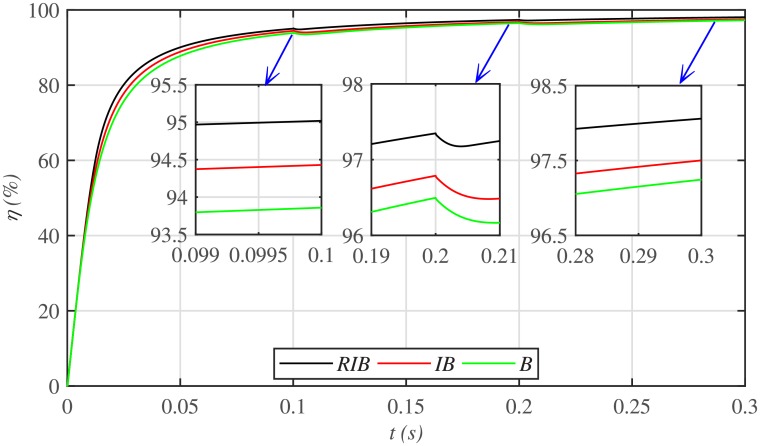
PV array efficiencies comparison under case 3.

Similarly, Fig 24 depicts the comparative plot for the PV array output powers under case 3. It can be observed that both the backstepping and integral backstepping controllers has the worst performance under plant parametric uncertainties. However, the proposed RIB control technique remains unaffected, achieves steady-state faster and offers a superior tracking performance. Moreover, the proposed technique renders the minimum tracking error as compared to the other two MPPT candidates as depicted in Figs 25 and 26. Similarly, Fig 27 shows that the proposed RIB based MPPT strategy has the highest efficiency than its competitors.

### 5.4 Comparison with conventional MPPT techniques

A comparative analysis between the proposed RIB control based MPPT scheme and the well-known conventional PID and P&O based MPPT techniques is presented in this section.

#### 5.4.1 Performance test against varying meteorological conditions

In this case study, a comparative analysis is presented between the proposed RIB based MPPT strategy and the traditional PID and P&O based MPPT techniques under the same scenario as discussed in case1.

**Fig 28:**
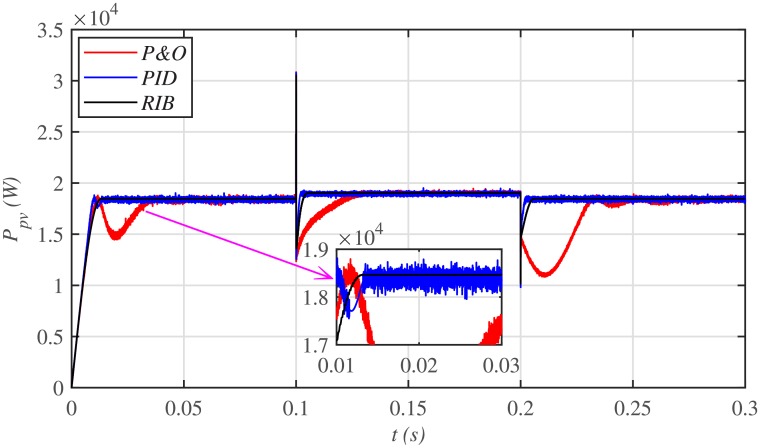
PV array comparative powers under varying meteorological conditions.

Fig 28 depicts the comparative plot for the PV array output powers. It can be seen that both the PID and P&O based MPPT techniques suffer from a lot of oscillations around the *V*_*MPP*_ under their steady-states. This is practically undesirable. However, the proposed RIB control technique performs the best with negligible steady-state error, thus, outperforming both the PID and P&O control techniques.

#### 5.4.2 Performance test against faults under varying meteorological conditions

In this case study, the proposed technique is further tested and validated by comparing its MPPT performance with the PID and P&O based MPPT control techniques under the same conditions as discussed in case 2.

**Fig 29:**
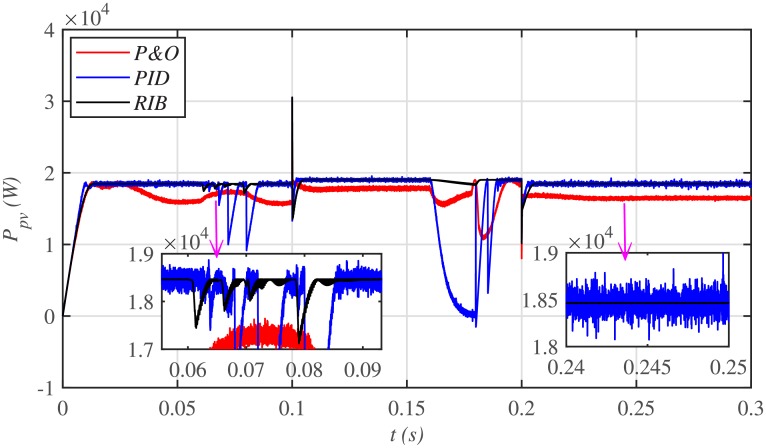
PV array comparative powers under faults and varying meteorological conditions.

It is evident from Fig 29, that both the conventional PID and P&O based MPPT control techniques deviate from *P*_*MPP*_ upon the occurrence of faults in the plant, while the proposed MPPT technique exhibits more robustness against plant faults with negligible deviation.

#### 5.4.3 Performance test against uncertainties under varying meteorological conditions

Finally, in this case study, a comparative analysis between the proposed RIB based MPPT control strategy and the conventional PID and P&O based MPPT control techniques is presented under the same conditions as discussed in case 3.

**Fig 30:**
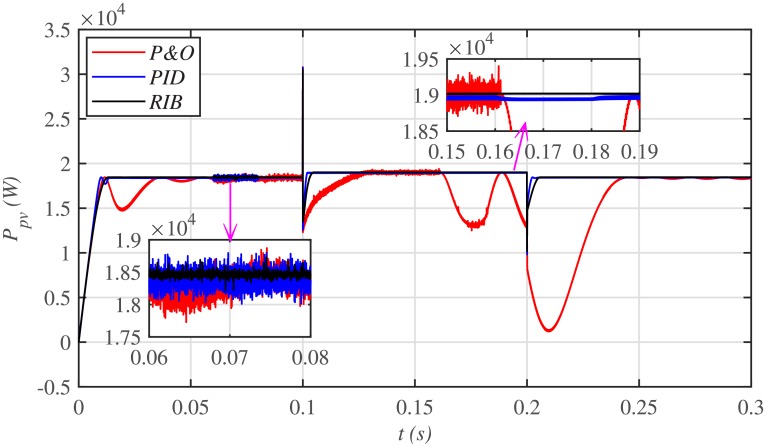
PV array comparative powers under uncertainties and varying meteorological conditions.

Fig 30 depicts the comparative plot for the PV array output powers. It can be observed that the proposed RIB control technique delivers the maximum power with the minimum chattering as compared to the PID and P&O based control techniques. It means, that the proposed technique is more robust to plant parametric uncertainties than the conventional MPPT candidates.

## 6 Conclusions and future research recommendations

This research paper presents a nonlinear, hybrid, very fast and efficient robust integral backstepping based MPPT control approach for a stand-alone PV system, consisting of a PV array, a non-inverting DC-DC buck-boost power converter, a purely resistive and an dynamic load. The DC-DC buck-boost converter is employed as a power electronic interface between the PV array and the load. The proposed MPPT control scheme consists of two loops, where the first loop generates the real-time offline reference peak power voltage through an ANFIS network, which is then utilized in the second loop as a set-point value for generating a control signal and then forcing the PV system to be operated at this set-point by continuously adjusting the duty ratio of the power converter. The superiority of the proposed MPPT approach is validated through Matlab simulations carried out under time varying plant faults, plant parametric uncertainties and varying meteorological conditions. The comparative analysis depicts that the proposed MPPT control strategy exhibits a superior tracking performance with no overshoot, fast dynamic response, less rising and settling times and minimum output tracking error as compared to the existing backstepping, integral backstepping and conventional PID and P&O based MPPT control schemes.

The proposed MPPT control paradigm successfully achieved the desired objectives under a standalone PV system. The authors are working to test and validate its effectiveness under grid-connected system and under partial shading conditions. Hopefully, it will be submitted separately for a review in near future.
